# Development of a Species Diagnostic Molecular Tool for an Invasive Pest, *Mythimna loreyi,* Using LAMP

**DOI:** 10.3390/insects11110817

**Published:** 2020-11-19

**Authors:** Hwa Yeun Nam, Min Kwon, Hyun Ju Kim, Juil Kim

**Affiliations:** 1 Highland Agriculture Research Institute, National Institute of Crop Science, Rural Development Administration, Pyeongchang 25342, Korea; jessienam89@gmail.com (H.Y.N.); mkwon@korea.kr (M.K.); 2 Crop foundation Division, National Institute of Crop Science, Rural Development Administration, Wanju 55365, Korea; yaehyunj@korea.kr; 3 Program of Applied Biology, Division of Bio-resource Sciences, College of Agriculture and Life Science, Kangwon National University, Chuncheon 24341, Korea

**Keywords:** *Mythimna loreyi*, rice armyworm, invasive pest, LAMP, diagnostic PCR

## Abstract

**Simple Summary:**

*Mythimna loreyi* is a serious pest of grain crops and reduces yields in maize plantations. *M. loreyi* is a native species in East Asia for a long time. However, this species has recently emerged as a migration (or invasive from other Asian countries) pest of some cereal crops in Korea. Little is known about its basic biology, ecology, and it is difficult to identify the morphologically similar species, *Mythimna separate*, which occur at the cornfield in the larvae stage. Species diagnosis methods for invasive pests have been developed and utilized for this reason. Currently, the molecular biology method for diagnosing *M. loreyi* species is only using the mtCO1 universal primer (LCO1490, HCO2198) and process PCR and sequencing to compare the degree of homology. However, this method requires a lot of time and effort. In this study, we developed the LAMP (loop-mediated isothermal amplification) assay for rapid, simple, and effective species diagnosis. By analyzing the mitochondrial (mt) genome, the species-specific sequence was found at the coding region of the NADH dehydrogenase subunit 5 gene. A broad range of DNA concentration was workable in LAMP assay, in which the minimum detectable DNA concentration was 100 pg. DNA releasing method was applied, which took five minutes of incubation at 95 °C without the DNA extraction process, and only some pieces of tissue from larvae and adult samples were needed. The incidence of invasive pests is gradually diversifying. Therefore, this simple and accurate LAMP assay is possibly used in the intensive field monitoring for invasive pests and integrated management of *Mythimna loreyi*.

**Abstract:**

The *Mythimna loreyi* (Duponchel) is one of the well-known invasive noctuid pests in Africa, Australia, and many Asian countries. However, it is difficult to identify the invasive and morphologically similar species, *Mythimna separate*, which occur at the cornfield in the larvae stage. Currently, the molecular biology method for diagnosing *M. loreyi* species is only using the mtCO1 universal primer (LCO1490, HCO2198), which requires a lot of time and effort, such as DNA extraction, PCR, electrophoresis, and sequencing. In this study, the LAMP assay was developed for rapid, simple, effective species identification. By analyzing the mitochondrial genome, the species-specific sequence was found at the coding region of the NADH dehydrogenase subunit 5 gene. Based on this unique sequence, four LAMP primers and two loop primers were designed. The F3 and B3 primers were able to diagnose species-specific, in general, and multiplex PCR and specifically reacted within the inner primers in LAMP assay. The optimal incubation condition of the LAMP assay was 61 °C for 60 min with four LAMP primers, though additional loop primers, BF and LF, did not significantly shorten the amplification time. The broad range of DNA concentration was workable in LAMP assay, in which the minimum detectable DNA concentration was 100 pg. DNA releasing method was applied, which took five minutes of incubation at 95 °C without the DNA extraction process. Only some pieces of tissue of larvae and adult samples were needed to extract DNA. The incidence of invasive pests is gradually diversifying. Therefore, this simple and accurate LAMP assay is possibly applied in the intensive field monitoring for invasive pests and integrated management of *Mythimna loreyi*.

## 1. Introduction

The *Mythimna loreyi* (Duponchel) (often called the cosmopolitan) is a noctuid pest of grain crops found in Africa, Australia, the Near East, and the Middle East and undergoes multiple generations per year [1,2,3,4]. *M. loreyi* feeds on various host plants, including rice, wheat, maize, sugarcane, barley, sorghum, and others, which have a large effect on female fecundity. The fecundity of female moths is greatest when the larvae feed on maize in Egypt [5]. Since *M. loreyi* is facilitated to breed, some researchers focus on the identification of products secreted by the adult corpora allata [6]. Not only the physiological understanding of this species [7,8,9,10] but also ecology-based developmental characteristics [11] and flight performance [12] have been studied. For the biological control, *M. loreyi* densovirus (MIDNVs) was isolated in Egypt and characterized [13,14]. The outbreak of this pest and the damage to crops have been proliferated, particularly in some Asian countries. In Japan, *M. loreyi* typically occurs together with *M. separate* (Walker) that has significant negative effects on crop production [15]. Besides, *M. loreyi* has begun to occur and damage host plants together with *M. separate* [16].

During a couple of years in 2019 and 2020, there have been reports that cornfield has been damaged by the larvae of *M. loreyi* in Korea. This indicates that there is a possibility that *M. loreyi* can change into a sporadic pest, which can cause serious damage to crops. As per these cases, *M. loreyi* is occasionally damaged with its sister species, *M. separate*. However, it is difficult to distinguish two species at the cornfield in the larvae stage. Only one molecular diagnostic tool has been studied to distinguish *M. loreyi,* which is based on the sequencing of part of the mitochondrial COI gene [17]. However, only one mutation exists within the 658 bp amplicon. Moreover, high sequence similarity has been shown between *M. loreyi* and *M. separata*, which indicates the limitation of diagnosis of this pest.

In this study, we developed a simpler technique, termed loop-mediated isothermal amplification assay (LAMP). It is also widely used for the rapid and accurate identification of pest species [18,19,20]. The LAMP is a rapid, simple, effective, and specific amplification of DNA compared to real-time PCR based on the mitochondrial gene. It is performed under isothermal conditions that require a set of four primers, a strand-displacing DNA polymerase, and a water bath or heat block to maintain the temperature at about 65 °C following a one-time denaturation at 95 °C [21] or one-step incubation at about 65 °C [22].

Following the first infestation of *M. loreyi* in Korea, there is great demand from agricultural research, extension services, and farmers for diagnostic methods for these species. Therefore, we present a LAMP-based method for specimens collected in Korea and other sequences from GenBank. This method should be useful in assisting the effective pest management of *M. loreyi*.

## 2. Materials and Methods

### 2.1. Sample Collection and Mitochondrial Genome Sequencing

The larval stage of *Mythimna loreyi* Korean populations was collected from Hadong (35°02′17″ N, 127°47′12″ E) in a cornfield, 2019. Some larvae reared in the lab for morphological conformation in the adult stage, and the genomic DNA of several of the individual larva was directly extracted with DNAzol (Molecular Research Center, Cincinnati, OH, USA) and quantified by Nanodrop (NanoDrop Technologies, Wilmington, DE, USA). Besides, the genomic DNA of over 20 larvae or adults was extracted (population pooled genomic DNA). Universal primers (LCO1490 and HCO2198) were used with *M. loreyi’s* individual and pooled genomic DNA as templates in 20 μL PCR reaction containing 1 U TOYOBO KOD—FX TaqTM (Toyobo Life Science, Osaka, Japan), 2X buffer (with 15 mM MgCl2), 0.2 mM each dNTP, 0.5 μM each primer, and 100 ng genomic DNA [23]. The PCR products were directly sequenced (chromatogram) to verify the nucleotide polymorphism, and no mutation was found in intraspecies. For mitochondrial genome sequencing, the Miseq platform was used, and more than 1 Gb was sequenced. To assemble these data, the CLC Assembly Cell package (version 4.2.1) was used. After trimming raw data using CLC quality trim (ver. 4.21), the assembly was accomplished using the CLC de novo assembler with dnaLCW. Assembled sequences were confirmed by BLASTZ [24]. The GeSeq program was used for annotation [25], and the result was manually checked based on the alignment of other Noctuidae species mitochondrial genomes using MEGA 7 [26].

### 2.2. Phylogenetic Analysis and Primer Design

Molecular phylogenetic analysis of mitochondrion genomes was inferred by using the maximum likelihood method implemented by MEGA 7 with bootstrapping [26,27]. Mitochondrial genome sequences of other Noctuidae species were downloaded from GenBank, NCBI. For comparative analysis, mitochondrial genomes were aligned using mVISTA [28,29]. Based on the global alignment result, partial sequences were re-aligned for LAMP primer design using PrimerExplorer V5.

### 2.3. LAMP and PCR

WarmStart^®^ LAMP Kit (New England Biolabs, Ipswich, UK) was used for the LAMP assay. The general protocol of LAMP was performed following the manufacture’s guidelines in a 25 μL reaction mixture. For the general PCR, TOYOBO KOD—FX TaqTM (Toyobo Life Science, Osaka, Japan) was used in this study. Appropriate primers were used with the following PCR amplification protocol: a 2 min denaturing step at 94 °C and a PCR amplification cycle consisting of denaturing at 94 °C for 20 s, annealing at 60 °C for 20 s, and extension at 68 °C for 30 s, which was repeated 35 times. The amplified DNA fragments were separated using 1.5% agarose gel electrophoresis and visualized with SYBR green (Life Technologies, Grand Island, NY, USA). The larval stage of *M. loreyi* samples, which were collected from Hadong (Korea) in a cornfield (2019), was used. Pheromone traps were used for adults’ sample collection, such as *M. separate*, *Agrotis segetum*, *Spodoptera frugiperda*, *Spodoptera exigua*, *Spodoptera litura*, and *Helicoverpa armigera*. Traps were set in Pyeongchang (37°40′53″ N, 128°43′49″ E), Hongchen (37°43′35″ N, 128°24′33″ E), and Gangneung (37°36′56″ N, 128°45′59″ E) [30]. DNA samples were prepared using DNAzol (Molecular Research Center, Cincinnati, OH, USA) from trapped adults. We used universal primers (LCO1490 and HCO2198) with each DNA sample and pooled genomic DNA as templates in a 20 μL PCR reaction containing 1 U TOYOBO KOD—FX TaqTM (Toyobo Life Science, Osaka, Japan), 2X buffer (with 15 mM MgCl2), 0.2 mM each dNTP, 0.5 μM each primer, and 100 ng genomic DNA [23]. The PCR products were directly sequenced to verify the mutation of each population using RAW_F3S and RAW_B3 primer set (MACROGEN). Three biological DNA samples that were collected from the fields were used in each LAMP and PCR to validate the reliability of the LAMP condition.

## 3. Results

### 3.1. Mitochondrial Genome Sequencing and Primer Design

A 15,320 bp of mitochondrial genome verified after trimming from about 2.2 Gb (7,312,504 reads) nucleotide sequences information was obtained through *Miseq.* The mitochondrial genome *of Mythimna loreyi* was assembled (GenBank MT506351). The mitochondrial genome included 13 protein-coding genes: NADH dehydrogenase components (complex *I*, ND), cytochrome oxidase subunits (complex VI, COX), cytochrome oxidase b (CYPB) and two ATP synthases, and two ribosomal RNA genes and 22 transfer RNAs (Supplementary Figure S1).

As a result of MegaBLAST, the most homologous species was an allied species, *Mythimna separate*, which showed 93.7% similarity. The genus of Spodoptera [31], such as *Spodoptera exigua*, *S. litura, S. frugiperda*, which are possibly found together with *M. loreyi* in the cornfields, showed about 89 to 90% homology based on the mitochondrial genome sequence. *Agrotis segetum* [32], which, in particular, occurs and damage at the corn seedling stage, showed 90.6% similarity to *M. loreyi* based on mitochondrial genome sequence (data not shown).

**Figure 1:**
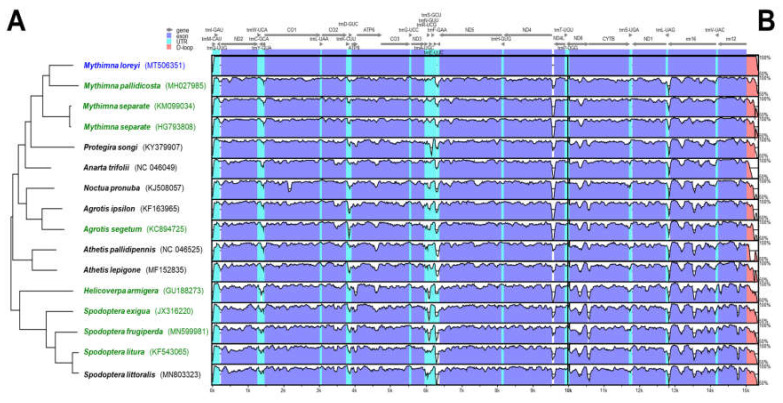
Comparison of entire mitochondrial genomes of some Noctuidae pests, including newly sequenced *Mythimna loreyi*. (**A**) Phylogenetic relationship inferred using maximum likelihood under MEGA7. (**B**) Schematic diagram of the genes and their flanking regions, showing the sequence diversity in mVISTA. UTR, D-loop denotes untranslated region and displacement-loop, respectively. Eight green colored mitochondrial genome sequences were re-aligned for primer design with that of the target species, *M. loreyi* (Figure 2).

**Figure 2:**
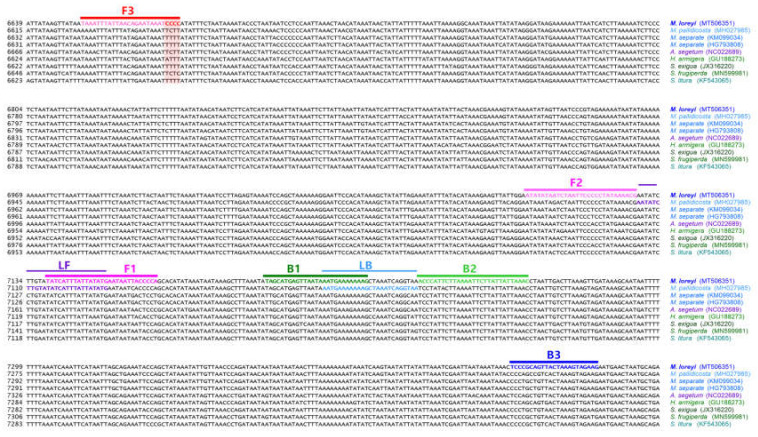
Location of primers and primer binding regions on partial sequence of some Noctuidae pests’ mtDNA for species identification of *M. loreyi*. Inner primer, FIP, consists of F1c (complementary sequences of F1) and F2. Another inner primer, BIP, is also composed of B1 and B2c (complementary sequences of B2). Essential four LAMP primers (F3, FIP, BIP, and B3) generate the dumbbell structure, and two loop primers, LF and LB, possibly accelerate the LAMP (loop-mediated isothermal amplification) reaction. Used primer information is documented in Table 1.

The phylogenetic relationship between 15 mitochondrial genomes of 14 species was examined (Figure 1A) to verify a specific nucleotide sequence that only *M. loreyi* possessed among the related species with high gene similarity or a morphologically similar pest. The result of the phylogenetic relationship was mostly similar to the megaBLAST result. Based on the mVISTA alignment results, conserved regions among Noctuidae species and variable regions were observed (Figure 1B). By combining the two results, the partial sequence in eight species of nine ND5 mitochondrial genome was re-aligned to design the specific primer of the *M. loreyi*. Finally, four essential primers and two loop primers were designed (Figure 2 and Table 1).

Among the six primers, F3 is the specific primer that enables the diagnosis of *M. loreyi*, and only *M. loreyi* had the CCCC sequences in four priming regions, which are marked in the red box. A total of four populations that were collected from different regions had the same nucleotide sequence. Therefore, it could be sufficiently used for species diagnosis. The basic species diagnosis primer production strategy is the same as the previously reported species diagnosis development method of *S. frugiperda* [33]. We targeted the open reading frame (ORF) region because the ORF region is more intraspecies conserved than the non-coding region.

### 3.2. Diagnostic LAMP and PCR

**Figure 3:**
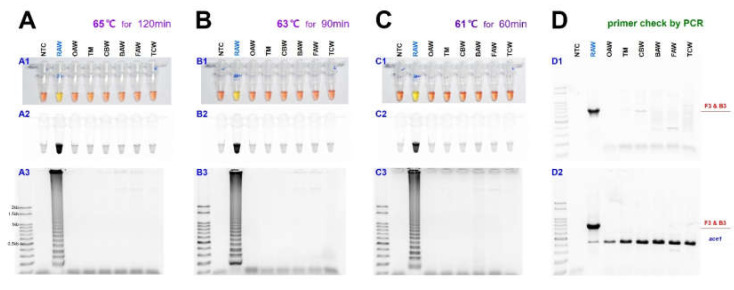
The sensitivity of the LAMP assay results in three temperature conditions, such as 65, 63, and 61 °C, for *M. loreyi* species detected under (**A1**, **B1**, and **C1**) visible light, (**A2**, **B2**, and **C2**) ultraviolet light with SYBR Green, and (**A3**, **B3**, and **C3**) gel electrophoresis. The original pink color of the reaction mixture turned yellow in a positive reaction when the product was formed but remained pink in negative reactions. (**D**) Conventional and multiplex PCR to distinguish *M. loreyi*. The 794 bp amplicon was amplified only in *M. loreyi,* and the conserved partial sequence of ace1 type acetylcholinesterase gene was targeted as an internal reference. Abbreviations are NTC (non-template control), RAW (rice armyworm *Mythimna loreyi*), OAW (oriental armyworm *Mythimna separata*), TM (Turnip moth *Agrotis segetum*), CBW (cotton bollworm *Helicoverpa armigera*), BAW (beet armyworm *Spodoptera exigua*), FAW (fall armyworm *Spodoptera frugiperda*), and TCW (tobacco cutworm *Spodoptera litura*).

As previously reported, the sensitivity of LAMP may vary depending on the temperature and reaction time [33]. Therefore, the reaction was performed at 65, 63, and 61 °C to find the optimal reaction conditions (Figure 3).

As the amount of template DNA was quantified as 50 ng and reacted at each temperature with 25 µL reaction volume, the diagnosis result was confirmed in 120 min at 65 °C and 90 min at 63 °C. The diagnostic level of reaction did not occur when the reaction performed less than the corresponding time in each temperature. Despite the relatively low temperature, at 61 °C, the diagnostic level of reaction was confirmed in only 60 min, and the false-positive reaction did not appear only once in the results of more than three repetition tests. Despite the relatively low temperature, at 61 °C, the diagnostic level of reaction was confirmed in only 60 min, and the false-positive reaction did not appear only once in three repetition tests (Figure 3C). Under 100 bp sized bands were not the LAMP products and were aggregated primers and generated even in the negative control.

In the LAMP assay, to confirm the diagnosis primer F3, it was reacted with reverse primer B3 through PCR, and the 794 bp of PCR product was identified (Figure 3(D1)). Besides, a species-specific reaction was confirmed, as we performed the multiplex PCR with a universal positive control primer set that targets ace1-type acetylcholinesterase, which can produce a positive reaction in all samples (Figure 3(D2)). The two-loop primers were tested for possible enhancement of the LAMP reaction, as suggested by Nagamine et al. [22].

**Figure 4:**
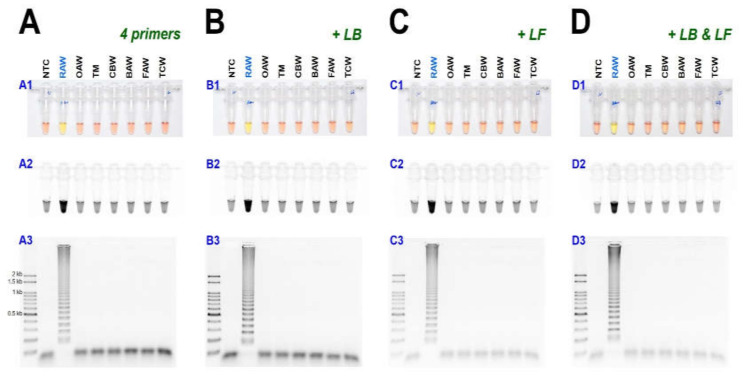
The LAMP assay results with (**A**) 4 primers and additional loop primers, (**B**) loop forward, LF, or (**C**) loop backward, LB, or (**D**) two-loop primers, LF and LB under (**A1**, **B1**, **C1**, and **D1**) visible light, (**A2**, **B2**, **C2**, and **D2**) ultraviolet light with SYBR Green, and (**A3**, **B3**, **C3**, and **D3**) gel electrophoresis. Abbreviations are NTC (non-template control), RAW (rice armyworm *Mythimna loreyi*), OAW (oriental armyworm *Mythimna separata*), TM (Turnip moth *Agrotis segetum*), CBW (cotton bollworm *Helicoverpa armigera*), BAW (beet armyworm *Spodoptera exigua*), FAW (fall armyworm *Spodoptera frugiperda*), and TCW (tobacco cutworm *Spodoptera litura*).

**Figure 5:**
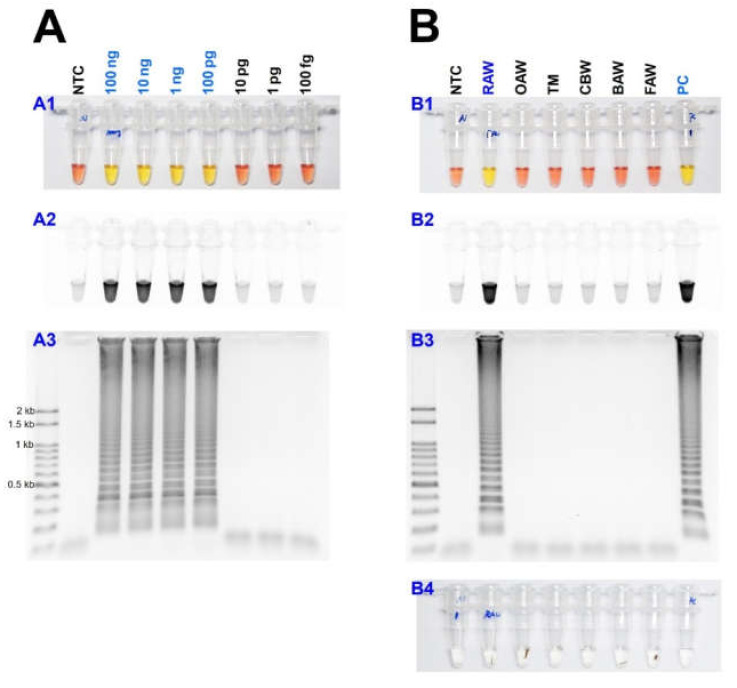
(**A**) Identification of the detection limit of genomic DNA in the LAMP assay from 100 ng to 100 fg under (**A1** and **B1**) visible light, (**A2** and **B2**) ultraviolet light with SYBR Green, and (**A3** and **B3**) gel electrophoresis. (**B**) The sensitivity of the LAMP assay results with the DNA releasing technique from insect tissue. Around 10 mg of the adult leg (or antenna) was incubated at 95 °C for 5 min (**B4**). Abbreviations are NTC (non-template control), RAW (rice armyworm *Mythimna loreyi*), OAW (oriental armyworm *Mythimna separata*), TM (Turnip moth *Agrotis segetum*), CBW (cotton bollworm *Helicoverpa armigera*), BAW (beet armyworm *Spodoptera exigua*), FAW (fall armyworm *Spodoptera frugiperda*), and PC (positive control, isolated DNA from *M. loreyi*).

As a result of reacting two-loop primers with each or two together at 61 °C for 60 min, there was no difference between the reaction result of using only four primers and shortening the reaction time (Figure 4). Even when 10 µL of the reaction solution was verified by electrophoresis after the LAMP reaction, there was no difference in band intensity. It was possible to reliably diagnose up to 100 pg when reacting using four LAMP primers without adding a loop primer (Figure 5A). Therefore, *M. loreyi* species diagnosis LAMP method that we developed could diagnose under various DNA concentration conditions from 100 ng to 100 pg. To increase the usability in the field, we cut a part of the tissue of the adult antenna or leg and put it in 30 µL distilled water, which reacts at 95 °C for 5 min, securing the template DNA without a separate DNA extraction process (Figure 5(B4)). As we measured each sample of DNA concentration obtained through this DNA releasing method, each sample showed various measurements. The species-specific diagnosis was possible in the same method as using the template DNA, which was obtained through separate DNA extraction as a positive control since it was in the range of LAMP reaction (Figure 5B).

## 4. Discussion

Invasive pests, such as *S. frugiperda*, are increasing worldwide due to global warming and climate change [34,35]. Currently, *M. loreyi* is a serious grain pest in Africa, Australia, the Near East, and the Middle East and undergoes multiple generations per year [1,2,3,4]. Besides, *M. loreyi* is a species that originates from China and results in damage upon spreading gradually. In Korea, *M. loreyi* has been reported for a long time and maybe a native pest to Korea. Recently, there are several reports that the larvae of *M. loreyi* have damaged the cornfield in Korea. There is a possibility that *M. loreyi* can change into a sporadic pest, which can cause serious damage to crops in Korea.

As with other invasive pests found in the field, they are often similar in morphology to allied species, making the investigation of initial density and pest management difficult. Species diagnosis methods for invasive pests, such as *S. frugiperda*, have been developed and utilized for this reason [33]. Currently, the molecular biology method for diagnosing *M. loreyi* species is only using the mtCO1 universal primer (LCO1490, HCO2198) and process PCR and sequencing to compare the degree of homology. However, this method requires a lot of time and effort, such as DNA extraction, PCR, electrophoresis, and sequencing. Therefore, we developed a molecular diagnosis method that could diagnose species within a short time without a separate DNA extraction process, and only a heat block is needed that can control temperature (Figure 5). The basis diagnosis strategy is very similar to the *S. frugiperda* species diagnosis method [33]. This method can complete all experimental procedures and verify the results within 1 h and 30 min right after obtaining a sample. In this study, the results only specified adult samples, but it is possible to use larvae.

The simplicity, accuracy, and adaptability for high throughput of the LAMP assay are distinct advantages [21,36,37]. Moreover, recently LAMP is utilized in various fields, such as many ecology studies, medical aspects, outside of the lab, and can be applied to diagnose plant viruses in insect body and insecticide-resistant gene mutation [38,39]. Besides, the diagnostic primer used for LAMP can be used for various diagnostic methods because it was possible to apply in general PCR and multiplex PCR (Figure 3). Therefore, it is feasible to diagnose a larger sample with positive control in the form of multiplex PCR, which is sufficiently modified and used in the laboratory. There are advantages and disadvantages to find a species diagnostic marker in the mitochondrial genome as well as in part of the genomic DNA. First of all, the disadvantage is that LAMP primer production is limited because there are many parts of AT-rich. It is complicated to design a primer that requires a minimum of four primers, and primers FIP and BIP are high-performance liquid chromatography (HPLC) purified primers. As an advantage, LAMP has higher amplification and efficiency and sensitivity compared to real-time PCR, and the results can be visually monitored by the naked eye either through turbidity or color change by fluorescent intercalating dye (Syber Green I) [40]. The number of copies is large, and it is possible to diagnose with a small amount of DNA or DNA releasing method, whose amplification can be accomplished with heating block. On the contrary, a species diagnosis marker in the genomic DNA should be found in exon rather than intron because it has a large variation. But it is difficult to develop marker in exon because it is often quite conserved within an allied species. Besides, the DNA releasing method can be used, but the efficiency is low when the copies of the gene are small [41].

## 5. Conclusions

In this study, a species diagnosis marker was designed within the mitochondrial genome to combine it with a DNA releasing method that is highly applicable to diagnose species in the field. Moreover, a significantly efficient method was developed, which targeted the ace1 gene as a positive control of the species to compare. This simple and accurate diagnosis using LAMP assay could be possibly applied in the intensive field to monitor and for the pest management of *M. loreyi*.

## Figures and Tables

**Table 1:** Primer list for LAMP (loop-mediated isothermal amplification) and PCR in this study.

Purpose	Primers	Sequence (5′– > 3′)
for LAMP		
	RAW_F3	GTAAATTTATTAACAGAATAAATCCCC ^1^
	RAW_B3	CTTCTACTTTAGTAACTGCGGGA
	RAW_FIP	TGGGGTAATTATTCATATAATAAATGATAATATATAATCTAATTCCCCCTATAAAACG
	RAW_BIP	TAGCATGAGTTAATAAATGAAAAAAAGGTTTAATAATAAGAATTTTAAGAATGGGT
	RAW_LF	CATATAATAAATGATATACAAGATATT
	RAW_LB	AATGAAAAAAAGCTAAATCAGGTAA
for PCR		
	Spo_ace1UF2	AGGATGAAGAGAAATTTATAGAGGAT
	Spo_ace1UR1	TCACCAAACACTGTATCTATAATTTG
	LCO1490	GGTCAACAAATCATAAAGATATTGG
	HCO2198	TAAACTTCAGGCTGACCAAAAAATCA
	RAW_F3S	CCCAAACCCTCTATATAATTCTCT

^1^ ‘G’s at the 5′ end depicted in red were added to adjust the primer melting temperature.

## Supplementary Materials

The following are available online at https://www.mdpi.com/2075-4450/11/11/817/s1, Figure S1: Organization of the mitochondrial genome of *Mythimna loreyi* from Korea (GenBank MT506351). ND: NADH dehydrogenase components (Complex I) in yellow. COX: cytochrome oxidase subunits (Complex VI) in pink. ATP synthase in green. CYPB: cytochrome oxidase b in purple. Ribosomal RNA genes in red, tRNA genes in blue. Noncoding regions are not colored.
