# Supplementary material for: Development of a Species Diagnostic Molecular Tool for an Invasive Pest, Mythimna loreyi, Using LAMP
Source: Insects. 2020 Nov 19;11(11):817. doi: 10.3390/insects11110817 (PMC7699485; doi:10.3390/insects11110817)
Supplement: Supplementary file 1 [file insects-11-00817-s001.pdf]

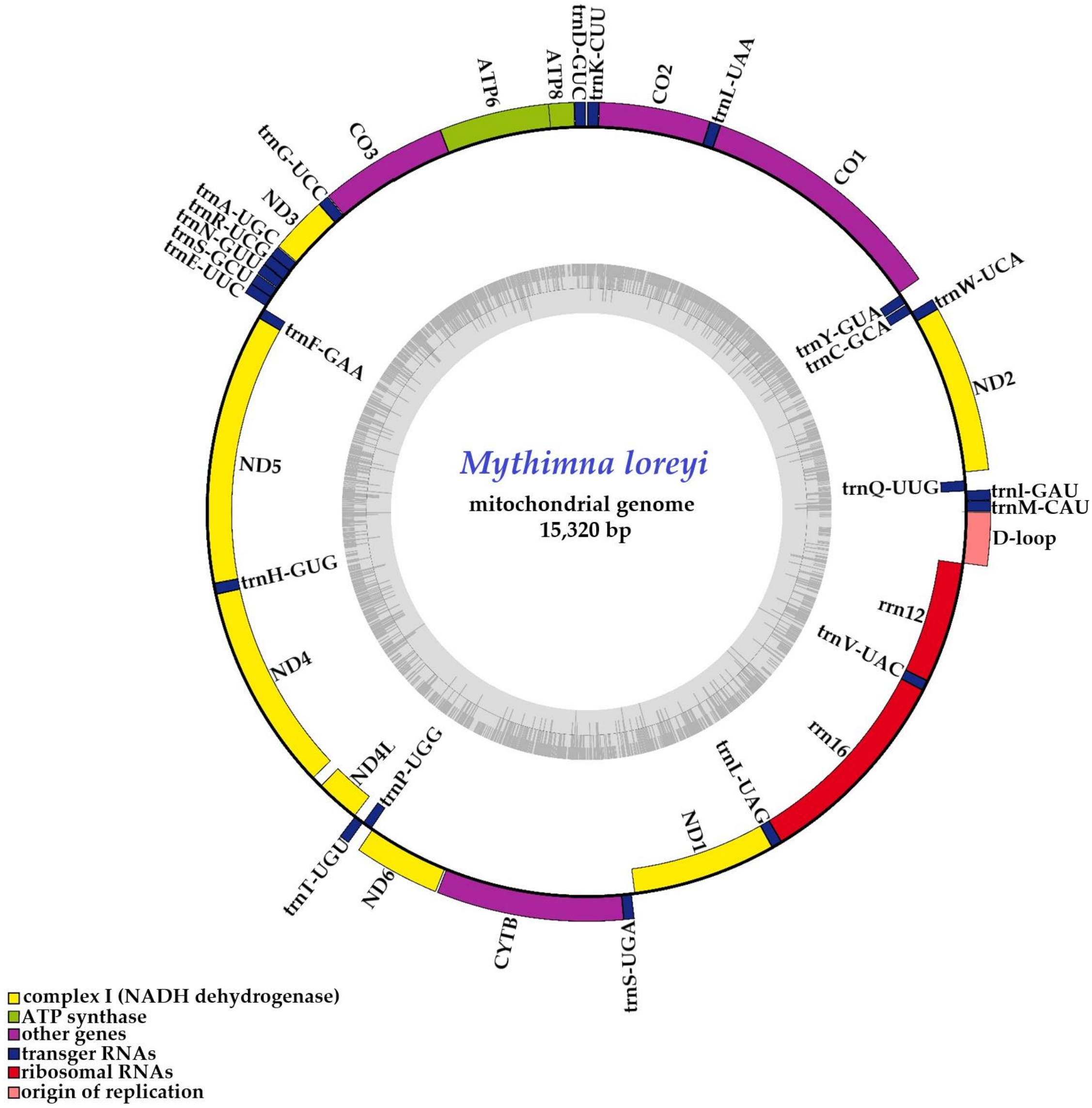

Figure S1. Organization of the mitochondrial genome of *Mythimna loreyi* from Korea (GenBank MT506351). ND: NADH dehydrogenase components (Complex I) in yellow. COX: cytochrome oxidase subunits (Complex VI) in pink. ATP synthase in green. CYPB: cytochrome oxidase b in purple. Ribosomal RNA genes in red, tRNA genes in blue. Noncoding regions are not colored.
